# Integration opportunities for HIV and family planning services in Addis Ababa, Ethiopia: an organizational network analysis

**DOI:** 10.1186/1472-6963-14-22

**Published:** 2014-01-18

**Authors:** James C Thomas, Heidi Reynolds, Christine Bevc, Ademe Tsegaye

**Affiliations:** 1 MEASURE Evaluation, Carolina Population Center and Department of Epidemiology, Gillings School of Global Public Health, University of North Carolina, Chapel Hill, USA; 2 MEASURE Evaluation, Carolina Population Center, University of North Carolina, Chapel Hill, USA; 3 North Carolina Institute for Public Health, Gillings School of Global Public Health, University of North Carolina, Chapel Hill, USA; 4 Management Sciences for Health, Addis Ababa, Ethiopia

**Keywords:** Social networking, Community health networks, Family planning services, HIV, AIDS

## Abstract

**Background:**

Public health resources are often deployed in developing countries by foreign governments, national governments, civil society and the private health clinics, but seldom in ways that are coordinated within a particular community or population. The lack of coordination results in inefficiencies and suboptimal results. Organizational network analysis can reveal how organizations interact with each other and provide insights into means of realizing better public health results from the resources already deployed. Our objective in this study was to identify the missed opportunities for the integration of HIV care and family planning services and to inform future network strengthening.

**Methods:**

In two sub-cities of Addis Ababa, we identified each organization providing either HIV care or family planning services. We interviewed representatives of each of them about exchanges of clients with each of the others. With network analysis, we identified network characteristics in each sub-city network, such as referral density and centrality; and gaps in the referral patterns. The results were shared with representatives from the organizations.

**Results:**

The two networks were of similar size (25 and 26 organizations) and had referral densities of 0.115 and 0.155 out of a possible range from 0 (none) to 1.0 (all possible connections). Two organizations in one sub-city did not refer HIV clients to a family planning organization. One organization in one sub-city and seven in the other offered few HIV services and did not refer clients to any other HIV service provider. Representatives from the networks confirmed the results reflected their experience and expressed an interest in establishing more links between organizations.

**Conclusions:**

Because of organizations not working together, women in the two sub-cities were at risk of not receiving needed family planning or HIV care services. Facilitating referrals among a few organizations that are most often working in isolation could remediate the problem, but the overall referral densities suggests that improved connections throughout might benefit conditions in addition to HIV and family planning that need service integration.

## Background

The needs of people infected with HIV and the needs of the organizations serving them are often misaligned. HIV infection affects virtually every biological and social system, each of which is intertwined with and affects the others. Few organizations are structured, however, to provide care in a holistic way reflecting these interdependencies. Instead, each is typically structured to address a specialized niche of care, such as the provision of medication, care for clinical symptoms, prevention of other infections, prevention of transmission to others, mental health, nutrition, family planning, or housing [1,2]. The specialization is driven in large part by the corresponding specialization of funding sources, be they departments in a ministry of health, non-governmental organizations (NGOs), foundations, or international donors. The specialization of the funders is driven, in turn, by the desire for accountability for the funds given, the ability to measure and count the number and types of services provided, and the ability of a particular organization to successfully attract funding. Family planning and HIV are a good example of services where historically the two have been largely siloed due to a number of factors including donor priorities [2].

The specialization and fragmentation of organizations is also manifested in geographical dispersion, so that organizations reduce their competition for the same client and/or attract underserved clients. Their competition for funds often results in service providers in the same community failing to work together to provide coordinated care for those with complex needs. This puts the burden on HIV-infected people to piece together a holistic response from a menu of specialized and geographically dispersed services. Studies have demonstrated that women living with HIV have unmet needs for contraception [3,4]; and that integration of family planning is feasible [3], can improve quality of care [5], and increase access to and uptake of services [2,4,6].

Health systems factors limit service delivery coordination and integration [7,8]. A recent review of the evidence of family planning and HIV service integration point out that most interventions are focused at the clinic level (training providers, use of job aids, etc.), and the authors conclude that there is a need to identify solutions at the broader health systems level [9]. Innovations are needed that shift some of responsibility from the provider-level to the organizational level, where organizations work together to meet the needs of their clients.

Apart from changing the incentives of organizations and the structures of funding streams, a move toward more holistic care for patients with complex needs can be achieved by giving organizations a new perspective on their place in a community and among the other service providers in the community. They can learn to see themselves not just as an isolated organization, but as a network of providers who have the potential to coordinate their efforts to meet the needs of the community. Further, they can see the potential for coordinating their care without the risk of losing their organizational identity. Coordination does not obviate competition. But neither does competition obviate coordination. Service providers can find opportunities to coordinate that provide benefits to both organizations and their service recipients. Research in the United States has shown that a higher level of organization interaction is associated with greater disease control [10,11].

Organizational network analysis (ONA) is a means of systematically collecting data on a set of organizations to describe quantitatively how they are connecting with each individually and as a whole. Through ONA, one can objectively describe the connections and discuss them with the organizations studied, enabling them to see the whole and decide whether there are redundancies or missed opportunities. The ONA also establishes a quantitative baseline that one can compare with over time, especially if the network is seeking change. We conducted two organizational network analyses in Addis Ababa, Ethiopia to identify the missed opportunities for the integration of HIV care and family planning service and to inform future network strengthening.

Addis Ababa, the capital city of Ethiopia, has a population of about four million, constituting roughly four percent of the country. The national prevalence of HIV is estimated at 1.5%, but residents of Addis Ababa are eight times more likely to be infected than those living in rural area [12]. Unintended pregnancies are still quite common in Ethiopia with 41% of all pregnancies being unintended in 2008 and a high of 72% in Addis Ababa [13]. In response to the burden of HIV, the unmet contraceptive needs, and the recognition for better coordination and networking between a broad range of service providers, the Ethiopian Ministry of Health and Federal HIV/AIDS Prevention and Control Office (HAPCO) identified strategic priorities for HIV prevention, care and treatment; one of which is referral linkages between service providers, including family planning services.

## Methods

In May 2011, we conducted this study in two of Addis Ababa’s ten sub-cities: Kirkos and Kolfe Keranyo. They were selected because of their similarities in population size, ethnic group make up, and proportions living in poverty. They are non-contiguous, thereby limiting the potential for organizational connections between the two sub-cities. Kolfe Keranyo, in the western portion of the city, has a population of over 260,000. It contains the city landfill and is generally thought to be the sub-city most affected by HIV. Kirkos is centrally located, with a population of more than 330,000. Both Kirkos and Kolfe Keranyo have large slum areas where many people living with HIV and clients of home based care services are concentrated.

### Data collection and management

The first step in the network analysis was to identify the relevant organizations in each sub-city. We included organizations that provided HIV care and support and/or family planning services to women living with HIV between the ages 18–49 living in Kirkos or Kolfe Keranyo. Beginning with the organizations known by HAPCO, we asked them what other organizations they knew of. In turn, we asked the same question of the newly named organizations. Eventually, no new organizations were named. We included in our network the organizations named by at least two other organizations. An exception to this rule was providers in private health clinics. Although considered by many to be a separate network for wealthier patients from the government/public sector network, we learned that private providers would occasionally refer a patient to a public clinic providing free HIV care. Because they were seldom named by the organizations serving low income patients, we included private clinics that were named only once.

Once the organizations were identified, we interviewed each of them about their interactions with each of the others. The respondents were senior organizational representatives, such as the manager or director, who were able to speak on a broad array of issues concerning the organization. They could bring into the interview any other staff they needed to help with the questions. The interviews, conducted by trained Ethiopian interviewers in the Amharic language, typically lasted about one hour.

The questionnaire was adapted from an instrument used by Thomas and colleagues in North Carolina [10] and South Africa (unpublished). It was translated into Amharic by an experienced English-Amharic translator; and back translated by the Ethiopian survey coordinator to confirm the intended meaning.

Questionnaire items were predominantly close-ended; responses to the few open-ended questions were later coded and categorized. The questions consisted of two general types of information: the organization’s attributes and its relations with other organizations. Attribute questions pertained to the organization’s services, staffing and clientele (Table 1). Specifically, information about the ‘type’ of organization was obtained in a self-reported response from the interviewee, the senior organizational representative, about their “type of facility, organization, bureau or office”. Information about median clients served, clinical and non-clinical staff, and clients per clinic or non-clinical staff was also obtained from what the organizational representative reported. Relational questions included the types of linkages (exchanges of clients [referrals], funds, other resources, or information) and their frequency. In this paper, we report on the linkages through client referrals.

**Table 1:** Attributes of the organizations in Kirkos and Kolfe Keranyo sub-cities of Addis Ababa, Ethiopia

**Organization attribute**	**Sub-city**	
	**Kirkos**	**Kolfe Keranyo**
Number		
HIV services only	18	5
Family planning services only	0	1
Both HIV and family planning services	7	20
Median		
Clients served^1^	219	1350
Clinical staff^2^	10	7
Non-clinical staff^3^	2	5
Clients per clinical staff	29	127
Clients per non-clinical staff	19	203

^1^In Kirkos, the mean was 726, median 220, with a range from 15 to 8,000. In Kolfe, the mean was 7,754, median 1,350, with a range from 99 to 150,000.

^2^Clinical staffing included medical doctors, health officers, nurses, nurse supervisors, lab technicians, and specialists (e.g. surgeon).

^3^Non-clinical staffing included health extension workers, community workers, as well as paid and unpaid volunteers.

Two Ethiopian research assistants participated in two days of training for this study. Both spoke fluent Amharic and English. The training included research ethics and pilot testing of the data collection instruments. All data were recorded on paper-based questionnaires. Two data entry specialists transferred the data into EpiData, a data management program; each double-entering the data to minimize miscoding. The data were then exported to R for analysis [14]. The study was approved by three ethics boards: the University of North Carolina, FHI 360 (who carried out the research through a subcontract with MEASURE Evaluation) and the Addis Ababa Health Bureau. The information obtained from organization respondents was neither personal nor private. We obtained verbal informed consent before conducting the interview.

### Network analysis

The networks in the two sub-cities were analyzed separately. Organizational attributes were described with univariate and bivariate statistics. Organizational relations were described in terms of overall network characteristics and an analysis of referral patterns.

We calculated several descriptive network characteristics, including network density (the number of links as a proportion of all those possible), centralization (the degree to which a high proportion of links were with a single or a select few organizations), and reciprocity (the proportion of ties that are mutual; e.g., A refers clients to B and B refers clients to A) [15]. To determine the frequency of client referrals between organizations, we calculated organization-level measures including in-degree (referrals received) and out-degree (referrals sent) [16].

Services can be “siloed”; organizations connect preferentially with others that are most like themselves [17-21]. For example, NGOs may connect with other NGOs rather than with government organizations. The network term for this is homophily, while the opposite tendency – preferential connections between dissimilar organizations - is heterophily [22]. Heterophily can occur when organizations with dissimilar services connect with each other. With exponential random graph (ERG) modeling we examined the probability of referral patterns between organizations based on their attributes, such as the type of organization, organization management, client to staff ratios, and driving distance [23-26] (available as Additional file 1). Descriptive and ERG model analyses were conducted with the *statnet* and *sna* packages of R [14,27].

A service gap was defined as an instance in which an organization could have but didn’t refer clients for services not provided by that organization. Of particular interest were gaps between HIV and family planning services. Gaps were identified visually using a grid that noted the nature of a given organization’s referrals to each other organization (available as an Additional file 2).

### Results interpretation workshop

In November 2011, the network results were presented to the network members during a one-day workshop in Addis Ababa. The purposes were to reveal to participants the network they were a part of, to give them an opportunity to comment on the findings, help interpret the data, and discuss how to improve the network connections. All of the organizations in the two sub-cities participating in the study were invited. Of the 51 organizations, 33 (65%) attended. They received a presentation of the study process and findings and two documents: a directory of the organizations in their network (the services each provided and the contact information) and a table listing the organizations that provide services that are complementary to theirs. Both were intended to facilitate future interactions between the organizations.

## Results

### Organization attributes

In Kolfe Keranyo, there were 26 organizations meeting the criteria for inclusion in our network analysis; in Kirkos, there were 25. All of them were successfully interviewed for our study. Although the numbers of organizations were similar in the two sub-cities, the types of organizations were different (Table 2).

**Table 2:** Types of organizations in Kirkos and Kolfe Keranyo sub-cities of Addis Ababa

**Organization type**	**Sub-city**	
	**Kirkos**	**Kolfe Keranyo**
Government hospital	0	1
Government health center	3	3
Government health post	0	3
Non-Governmental Organization (NGO)	14	6
Faith-Based Organization (FBO)	6	1
Private clinic	2	10
Other (Private hospital)	0	2
Total	25	26

Four out of five in Kirkos were civil society organizations: 14 were NGOs and 6 were faith-based. The only government organizations were 3 health centers. In contrast, the organizations in Kolfe Keranyo were more equally distributed among the government, civil society, and private health clinics. Type of organization was based on how the respondent characterized the type of organization during the interview.

Almost three quarters (72%) of the organizations in Kirkos offered only HIV-related services; the remainder offered both HIV and family planning services (Table 1). The opposite was true in Kolfe Keranyo: Three quarters (77%) offered both types of services and a minority offered services only for HIV or family planning.

Over the course of a month, organizations in Kirkos served fewer clients than the organizations in Kolfe Keranyo (medians of 219 and 1,350, respectively) (where the number of clients served is based on the respondent’s report of the number of clients served per month on average) (Table 1). Kirkos organizations reported a higher median number clinical and non-clinical staff than did Kolfe Keranyo. With fewer clients and more staff, the ratios in Kirkos were more favorable than in Kolfe Keranyo. The difference was due in part to the government hospital in Kolfe Keranyo which reported 150,000 clients. The government hospital was a general hospital; there was no government general hospital in Kirkos.

### Network characteristics

Although the two networks were composed of similar numbers of organizations, there were fewer referral ties in Kirkos (69) than in Kolfe Keranyo (101) (Table 3). This is reflected in the network densities (0.115 and 0.155, respectively). Organizations in Kirkos reported lower average numbers of referrals to other organizations (out-degree) and received referrals from other organizations (in-degree) than did organizations in Kolfe Keranyo. The organizations in both Kirkos and Kolfe Keranyo had a high proportion of mutual referralsbetween each other (network reciprocity). The Kirkos network was less centralized than the Kolfe Keranyo network, in large part because of the numerous ties to the government hospital in Kolfe Keranyo.

**Table 3:** Relational characteristics of the organizational networks in Kirkos and Kolfe Keranyo sub-cities of Addis Ababa, Ethiopia

**Network characteristic**	**Sub-city**	
	**Kirkos**	**Kolfe Keranyo**
Links	69	101
Density	0 .115	0.155
Centralization	0.192	0.525
Referrals sent (“out-degree”)^1^	2.76	3.88
Referrals received (“in-degree”)^2^	2.76	3.88
Reciprocity	0.83	0.78

^1^For Kirkos, the median for out-degree was 2, with a range of 0–8. For Kolfe, a median out-degree of 4, with a range of 1 to 9.

^2^For Kirkos, the median in-degree was 3, with a range of 0 to 7. For Kolfe, the median in-degree was 2.5, with a range of 0 to 23.

### Referral patterns

**Figure 1:**
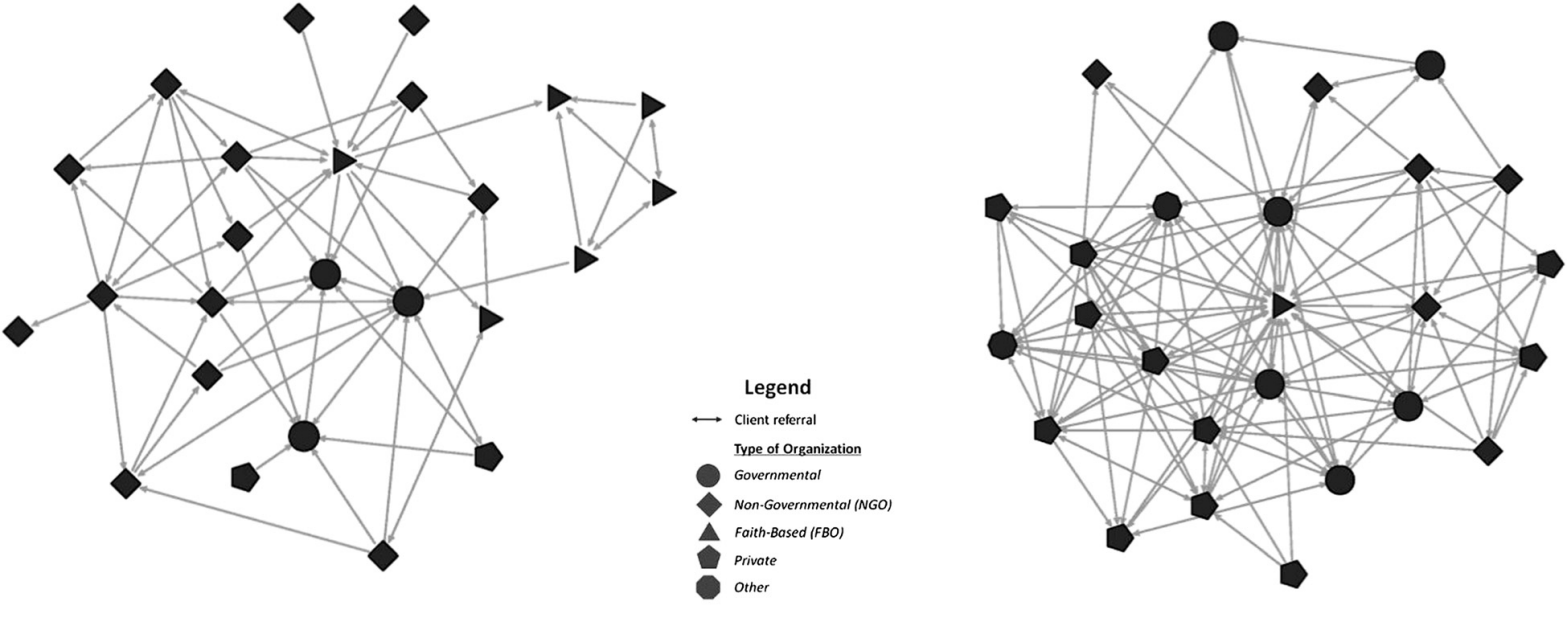
Client referrals among organizations in Kirkos (left) and Kolfe Keranyo (right) sub-cities of Addis Ababa, Ethiopia, May 2011.

**Figure 2:**
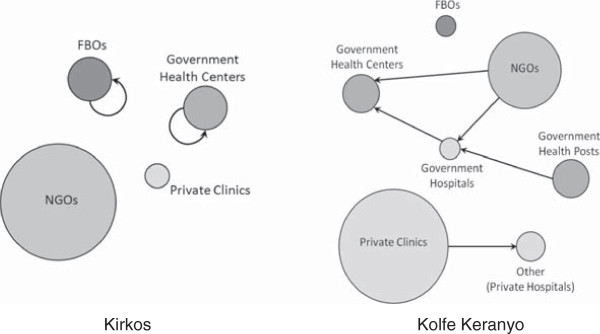
**Summary of dominant referral patterns among organization types in Kirkos and Kolfe Keranyo sub-cities of Addis Ababa, Ethiopia.** (Circle size is weighted to the number of organizations in the network for each type. An absence of arrows does not infer an absence of links).

The level of referrals wasn’t associated in either sub-city with the staff:client ratio or the driving distance between organizations (see online additional files). The patterns of connections between organizations were quite different, reflecting the different array of organizations in the two sub-cities (Figure 1). Faith-based organizations (FBOs) and government health centers in Kirkos tended to send clients to organizations similar to themselves (Figure 2 and online additional files). For example, a cluster of four FBOs formed a clique in which each referred to each of the others but only one of them referred to an organization outside of the clique (Figure 2). Organizations managed by a board, as many NGOs are, seldom referred clients to other organizations managed by a board. This could happen, for example, if NGO boards discourage client referrals to other NGOs.

The organizations in Kolfe Keranyo more often referred clients between organization types. The government health posts tended to refer clients to the government hospital, who in turn had a tendency to send clients to the government health centers. NGOs tended to send clients to both government hospitals and health centers, and private clinics were more likely to refer clients to other organizations, such as private hospitals.

Both HIV and family planning services were offered in just over one quarter of the organizations in Kirkos but over three quarters of the organizations in Kolfe Keranyo. Few organizations provided a full array of HIV services; thus, most would have to refer clients to ensure their access to comprehensive care.

Of the 18 organizations in Kirkos providing HIV but not family planning services, 16 referred at least one client to an organization providing family planning services, but two referred none. Only six organizations in Kolfe-Keranyo did not provide any family planning services; all of them referred at least some clients to an organization providing those services.

### Comments from the organizations

During the results interpretation workshop, the organization representatives affirmed their accuracy and expressed that connections between organizations were too few. Reasons offered for isolation included no opportunities to know people from other organizations, high staff turn-over, no mechanism for feedback from organizations when clients are referred, and competition between organizations to serve more clients. They suggested the creation of an inter-organizational referral feedback system, memoranda of understanding between organizations, opportunities for organization representatives to meet and share experiences, workshops on how to network effectively, and the identification of new connections in which both organizations could benefit.

## Discussion

We found two organizational networks of nearly identical size providing services for HIV care and family planning in two sub-cities of Addis Ababa. However, there were few additional similarities. In one network, a government hospital and associated clinics figured prominently. The other, lacking a hospital and with fewer government clinics, had more NGOs and FBOs and was less centralized.

We are unaware of any other publications reporting on HIV- or family planning-related organizational networks in developing countries. The only networks for comparison are in the US. A network of 30 HIV/AIDS services in Baltimore in 1997 had a referral density of 0.43 [28]. Thomas and colleagues measured the densities of information exchanges among HIV prevention organizations in ten counties of North Carolina [11]. The six counties with a high rate of syphilis (greater than 4.5 cases/100,000 population) had a median density of 0.156. The four counties with a low rate of syphilis (less than 3.5 cases/100,000) had a median density of 0.384. The higher densities in low rate counties suggest that better coordination may contribute to better disease control.

### Evaluating networks

Compared to the Baltimore network, the referral densities in the two Addis Ababa sub-cities are relatively low. The Baltimore connections may have existed in part from a donor program requiring a collaborative approach [28]. There were organizations in both sub-cities that provided care for people with HIV but did not attend to their family planning needs and were not referring their clients to other organizations that could. The opposite was also evident, though less clearly so since the family planning organizations also provided some HIV services. When they provided only a few HIV services and did not refer clients to other HIV organizations, one can infer that some HIV services were not being received, or the organization in question was not facilitating access to them.

Although the referral densities of the two sub-city networks were similar to each other on an absolute scale, they were composed of different organization mixes. Each network arose from a particular context in response to local needs and opportunities; each resulting structure has strengths and weaknesses. Hypothetically, a more centralized network, as in Kolfe-Keranyo, can afford quicker action and more coordination when the central organization dictates changes or procedures. However, if the central organization encounters a crisis, such as a loss of funding, negative consequences can ripple out to the rest of the network quickly. Centralized networks can thus be more efficient, but they are also more dependent on the success of the central organization.

A more decentralized network, as in Kirkos, has several organizations that are well connected but none that dominates. Should one of these encounter a crisis, the others could keep the network functioning. Decentralized networks, then, might not be as efficient as centralized ones but they could exhibit more resilience to shocks.

Our data offer little basis for determining whether either network is right; that it offers the optimal mix of services or the organizations coordinate in ways that best serve the clients’ needs. There is no gold standard for the density of referrals. More is not necessarily better. In some instances, referrals can be unnecessary and inefficient. Moreover, even though an outsider can point to organizations that could refer to each other based on the services offered, there may be reasons not captured by a questionnaire that would argue against those referrals. We learned in our presentation of results for example, that one organization to which others were referring was overwhelmed with received referrals and needed to decrease them.

In cultural anthropology, an emic perspective is that of cultural insiders. It stands in contrast to the etic perspective of outsiders. What a network should be depends largely on the emic perspective of providers and their clients. When providers of HIV care and reproductive health in Addis Ababa see objective documentation of how they are interacting, they need to address a number of questions.

Among them are the following: Is this the mix of services needed? Are clients’ needs being met? Are they connecting as you, the providers, would like them to? Are they connecting as the clients would like them to? What are the barriers to connection? Can they be addressed? With the insiders’ answers to questions such as these, insiders and outsiders alike will be better able to move the network towards the insiders’ ideal.

The etic and systematic perspective provided by the network sociogram and the gap analysis is a necessary complement to the insiders’ views. It enables the service providers to see themselves as a network when they are more naturally inclined to think about themselves as discrete organizations. And when they see the network, the data can guide them through questions about their connections and inform their desires for change.

### Network analysis limits

There are limits, of course, to the network perspective we describe. A single organization can be part of several networks at the same time. A medical clinic, for example, would likely address health concerns beyond HIV care or family planning, such as mental health or diabetes. There will be other organizations that it refers clients to for services it doesn’t provide itself; organizations that were not part of the network we described for HIV care and family planning. The HIV care and family planning network information provides limited guidance for those other connections.

We also did not ask respondents about referrals to or from organizations outside of their sub-city. For example, we did not ask organizations in Kirkos about referrals to the government hospital in Kolfe- Keranyo. The organizations to include in a network analysis depend on the question at hand. In our case, it was how organizations with similar or complementary missions located within the same sub-city interact with each other. Nor do we have information from community members about how they view the organizational network or how their behavior affects the network.

Our analysis presents a picture of the network at one particular point in time. Networks are inherently dynamic, constantly adapting to new challenges and opportunities. The network we described could be significantly different a few months later. The relevance of our analysis, however, was strengthened by the stakeholders’ view that the results reflected their experience. The results were thus helpful for discussing desired changes. In a forthcoming publication we will report the results of an intervention that implemented their recommendations.

## Conclusions

Because of gaps in referrals, women receiving HIV care in Kirkos were at risk of not receiving needed family planning services; and women receiving family planning services in either sub-city were at risk of not receiving needed HIV services. The gaps could be remediated with organizations adding more services, or by referring to more organizations. One could seek to increase referrals among a few organizations that are most often working in isolation. However, the low overall referral densities suggest there may be a prevailing culture or a health system that work against inter-organizational connections in general. Addressing the underlying reasons would likely benefit conditions in addition to HIV and family planning that need service integration. Whether focused to particular services or across sectors, organizational network analysis provides a systematic way to identify existing and potential connections.

## Abbreviations

NGO: Non-governmental organization; HAPCO: HIV/AIDS Prevention and control office of the Ethiopian ministry of health ERG, exponential random graph; FBO: Faith-based organization.

## Competing interests

The authors declare that they have no competing interests.

## Authors’ contributions

JT conceived the study, directed the analysis, interpreted the study results, and drafted the manuscript. HR contributed to the study conception, data analysis, results interpretation and writing of the manuscript. CB contributed to the data analysis, results interpretation and writing of the manuscript. AT contributed to the data acquisition, and revising the manuscript. All authors have read and approved the final manuscript.

## Authors’ information

JT is Director of MEASURE Evaluation, the United States Agency for International Development’s (USAID’s) flagship project for monitoring and evaluation of the implementation of its global health agenda. He is also author of *Epidemiological Methods for the Study of Infectious Diseases*, published by Oxford University Press. As such, he is one of the foremost authorities on evaluation in global health.

## Pre-publication history

The pre-publication history for this paper can be accessed here:

http://www.biomedcentral.com/1472-6963/14/22/prepub

## Supplementary Material

Additional file 1 ERGM results. Click here for file

Additional file 2 Kirkos Kolfe opportunities. Click here for file
