# Supplementary material for: Integration opportunities for HIV and family planning services in Addis Ababa, Ethiopia: an organizational network analysis
Source: BMC Health Serv Res. 2014 Jan 18;14:22. doi: 10.1186/1472-6963-14-22 (PMC3923232; doi:10.1186/1472-6963-14-22)
Supplement: Additional file 1 — ERGM results. [file 1472-6963-14-22-S1.pdf]

Results for eight exponential random graph models (M_1-M_8) of networks of HIV and family planning organizations in two sub-cities of Addis Ababa, Ethiopia in May 2011.

	**Kirkos**		**Kolfe Keranyo**	
	MLE (s.e.)	*Z-score*	*MLE (s.e.)*	*Z-score*
M_1: Uniform homophily, based on organization type	**0.521 (0.213) ***	**-11.963**	0.416 (0.219)	1.902
M_2: Differential homophily, based on organization type				
FBO	**0.847 (0.423) ***	**2.002**	n/a	n/a
Government HC	0.084	0.076	16.989 (629.897)	0.027
Government Hospital	--	--	n/a	n/a
Government HP	--	--	-0.304 (1.101)	-0.276
NGO	**0.490 (0.226) ***	**2.169**	0.116 (0.445)	0.261
Other	--	--	16.208 (738.503)	0.022
Private Clinic	n/a	n/a	0.237 (0.265)	0.895
M_3: Assortative mixing, based on organization type				
FBO → *FBO*	1.273 (0.729)	1.745	n/a	n/a
Government HC → *FBO*	0.041 (0.967)	0.042	n/a	n/a
Government Hospital → *FBO*	--	--	17.635 (2399.545)	0.007
Government HP → *FBO*	--	--	0.376 (1.248)	0.301
NGO → *FBO*	0.594 (0.674)	0.881	1.069 (0.852)	1.256
Other → *FBO*	--	--	n/a	n/a
Private Clinic → *FBO*	1.427 (0.865)	1.650	n/a	n/a
FBO Government HC	1.165 (0.806)	1.445	0.376 (1.248)	0.301
Government HC → *Government HC*	0.511 (1.254)	0.407	18.752 (1712.238)	0.011
Government Hospital → Government HC	--	--	18.229 (1863.921)	0.010
Government HP → Government HC	--	--	0.376 (.747)	0.503
NGO → Government HC	0.511 (0.738)	0.692	0.617 (0.541)	1.142
Other → Government HC	--	--	-0.540 (1.122)	-0.482
**Private Clinic → Government HC**	n/a	n/a	**0.936 (0.439) ***	**2.134**
FBO → Government Hospital	--	--	17.635 (2399.545)	0.007
Government HC → Government Hospital	--	--	18.229 (1863.921)	0.010
Government Hospital → Government Hospital	--	--	n/a	n/a
Government HP → Government Hospital	--	--	1.762 (1.248)	1.412
**NGO → Government Hospital**	--	--	**2.679 (1.122) ***	**2.388**
Other → Government Hospital	--	--	17.972 (2007.458)	0.009
Private Clinic Government Hospital	--	--	19.188 (1648.956)	0.012
FBO → Government HP	--	--	0.376 (1.248)	0.301
Government HC → Government HP	--	--	1.292 (0.713)	1.812

Government Hospital → Government HP	--	--	0.376 (1.248)	0.301
Government HP → Government HP	--	--	-0.540 (1.122)	-0.482
NGO → Government HP	--	--	-0.540 (0.677)	-0.798
Other → Government HP	--	--	n/a	n/a
**Private Clinic → Government HP**	--	--	**-2.298 (1.045) ***	**-2.198**
FBO → NGO	0.423 (0.681)	0.620	-0.540 (1.122)	-0.482
Government HC → NGO	0.119 (0.775)	0.153	-1.010 (0.788)	-1.282
Government Hospital → NGO	--	--	0.376 (0.899)	0.418
Government HP → NGO	--	--	-0.540 (0.677)	-0.798
NGO → NGO	0.916 (0.636)	1.441	-0.120 (0.495)	-0.243
Other → NGO	--	--	n/a	n/a
**Private Clinic → NGO**	0.000 (0.864)	0.000	**-2.298 (0.759) ****	**-3.029**
FBO → Other	--	--	n/a	n/a
Government HC → Other	--	--	-0.540 (1.122)	-0.482
Government Hospital Other	--	--	1.069 (1.435)	0.745
Government HP → Other	--	--	n/a	n/a
NGO → Other	--	--	-1.329 (1.072)	-1.239
Other → Other	--	--	17.972 (2007.458)	0.009
**Private Clinic → Other**	--	--	**1.475 (0.516) ****	**2.855**
FBO → Private Clinic	-0.278 (1.210)	-0.229	n/a	n/a
**Government HC → Private Clinic**	0.511 (1.254)	0.407	**-1.570 (0.771) ***	**-2.037**
Government Hospital → Private Clinic	--	--	0.222 (0.731)	0.303
Government HP → Private Clinic	--	--	-1.128 (0.655)	-1.723
**NGO → Private Clinic**	n/a	n/a	**-1.570 (0.571) ****	**-2.748**
Other → Private Clinic	--	--	-0.029 (0.570)	-0.052
Private Clinic → Private Clinic	n/a	n/a	n/a	n/a
M_4: Assortative mixing, based on HIV/FP service provision				
Both → Both	-0.156 (0.417)	-0.374	-0.466 (0.469)	-0.963
FP → Both	--	--	-0.228 (0.483)	-0.337
HIV → Both	-0.437 (0.285)	-1.534	-0.647 (0.677)	-1.226
Both → FP	--	--	**-2.325 (1.128) ***	**-2.062**
FP → FP	--	--	n/a	n/a
HIV → FP	--	--	-0.767 (1.212)	-0.633
Both → HIV	-0.501 (0.290)	-1.726	**-1.116 (0.546) ***	**-2.043**
FP → HIV	--	--	-0.767 (1.212)	-0.633
HIV → HIV	n/a	n/a	n/a	n/a
M_5: Node covariance, based on client-to-staff ratio	1.277^{-4} (7.406^{-5})	0.172	**-8.686^{-5} (3.542^{-5}) ***	**-2.452**
M_6: Node covariance, based on client-to-clinical staff ratio	-1.659^{-4} (7.180^{-5})	-0.231	**-4.740^{-5} (2.009^{-5}) ***	**-2.359**

M_7: Node covariance, based on client-to-non-clinical staff ratio	-9.580^{-5} (1.063^{-5})	-0.901	**-2.472^{-5} (1.035^{-5}) ***	**-2.388**
M_8: Edge covariance, based on driving distance	5.500^{-5} (3.790^{-5})	1.452	-1.650^{-5} (2.520^{-5})	-0.656

Statistical significance codes: '***' $\leq$ 0.001; '**' $\leq$ 0.01; '*' $\leq$ 0.05.

Condensed Summary of ERGM Analysis Results for Network Models (M_1-M_8)

	Kirkos		Kolfe Keranyo	
	MLE (s.e.)	*Z-score*	*MLE (s.e.)*	*Z-score*
M_1: Uniform homophily, based on *organization type*	0.521 (0.213) *	-11.963	0.416 (0.219)	1.902
M_2: Differential homophily, based on *organization type*				
FBO	0.847 (0.423) *	2.002	n/a	n/a
NGO	0.490 (0.226) *	2.169	0.116 (0.445)	0.261
M_3: Assortative mixing, based on organization type				
Private Clinic $\rightarrow$ Government HC	n/a	n/a	0.936 (0.439) *	2.134
NGO $\rightarrow$ Government Hospital	--		2.679 (1.122) *	2.388
Private Clinic $\rightarrow$ Government HP	--		-2.298 (1.045) *	-2.198
Private Clinic $\rightarrow$ NGO	0.000 (0.864)	0.000	-2.298 (0.759) **	-3.029
Private Clinic $\rightarrow$ Other	--		1.475 (0.516) **	2.855
Government HC $\rightarrow$ Private Clinic	0.511 (1.254)	0.407	-1.570 (0.771) *	-2.037
NGO $\rightarrow$ Private Clinic	n/a	n/a	-1.570 (0.571) **	-2.748
M_4: Assortative mixing, based on HIV/FP service provision				
Both $\rightarrow$ FP	--	--	-2.325 (1.128) *	-2.062
Both $\rightarrow$ HIV	-0.501 (0.290)	-1.726	-1.116 (0.546) *	-2.043
M_5: Node covariance, based on *client-to-staff ratio*	1.277^{-4} (7.406^{-5})	0.172	-8.686^{-5} (3.542^{-5}) *	-2.452
M_6: Node covariance, based on *client-to-clinical staff ratio*	-1.659^{-4} (7.180^{-5})	-0.231	-4.740^{-5} (2.009^{-5}) *	-2.359
M_7: Node covariance, based on *client-to-non-clinical staff ratio*	-9.580^{-5} (1.063^{-5})	-0.901	-2.472^{-5} (1.035^{-5}) *	-2.388
M_8: Edge covariance, based on *driving distance*	5.500^{-5} (3.790^{-5})	1.452	-1.650^{-5} (2.520^{-5})	-0.656

Non-significant results were excluded from this table.
Signif. codes: 0 '***' 0.001 '**' 0.01 '*' 0.05 '.'
"n/a" represents those measure
