# Supplementary material for: Integration opportunities for HIV and family planning services in Addis Ababa, Ethiopia: an organizational network analysis
Source: BMC Health Serv Res. 2014 Jan 18;14:22. doi: 10.1186/1472-6963-14-22 (PMC3923232; doi:10.1186/1472-6963-14-22)
Supplement: Additional file 2 — Kirkos Kolfe opportunities. [file 1472-6963-14-22-S2.pdf]

Opportunities and Options for the Integration of HIV and Family Planning Services

Review of Client Referrals and Services among Organizations

Kirkos

	ID	1	2	3	4	5	6	7	8	9	10	11	12	13	14	15	16	17	18	19	20	21	22	23	24	25
No. of HIV Services Provided		6	13	7	4	14	3	6	8	13	15	10	7	6	8	8	6	6	7	5	4	2	12	6	6	9
Offer Family Planning Services		No	No	No	No	**Yes**	**Yes**	No	No	**Yes**	**Yes**	No	No	**Yes**	No	No	No	No	No	No	**Yes**	No	**Yes**	No	No	No
No	1	-		●		○	○			○	○			○		●					○		○			
No	2		-	•		○	●			○	○		●	○	●		●				●	●	○			●
No	3	●	•	-	•	●	○			○	●			○	•	•	●				○	•	○			
No	4		●	•	-	○	○			●	●			○			•				○	•	•			
**Yes**	**5**	◦	◦	•	◦	-		◦	◦	●	●	◦	◦		◦	◦	◦	◦	◦	◦		•	•	◦	◦	•
**Yes**	**6**	◦	◦	◦	◦	•	-	◦	◦			◦	◦		◦	◦	◦	◦	◦	◦	•	◦		◦	◦	◦
No	7					○	○	-		●	◦			○		●					○	•	●			
No	8					○	○		-	◦	◦	•		○					●		○		•	●		
**Yes**	**9**	◦	◦	◦	◦			◦	◦	-		◦	◦		◦	◦	◦	◦	◦	◦		◦		◦	◦	◦
**Yes**	**10**	◦	◦	◦	◦	●		◦	◦	●	-	◦	◦		●	●	●	●	◦	◦		◦		◦	◦	◦
No	11					○	○		•	◦	◦	-		○					•		○		○	•		
No	12					•	○			◦	◦		-	○							○	●	•			•
**Yes**	**13**	◦	◦	◦	◦	●		◦	◦			◦	•	-	◦	◦	◦	◦	◦	◦		•		◦	◦	◦
No	14					●	○			●	●		●	○	-		•				•		●			•
No	15					○	○			○	○			○		-					○		●			
No	16		•	•	●	•	○			○	•			○	●		-				○	•	○			
No	17					●	○			●	●			○				-			○		○			
No	18					○	○		●	○	○	●		○					-		○		•	●		
No	19	•				○	○			○	•			○						-	○		●		•	
**Yes**	**20**	◦	●	◦	◦			●	◦	●	●	◦	●		•	◦	◦	◦	◦	◦	-	•	●	◦	◦	•
No	21		●			○	○			○	○			○	•	•	•				●	-	●			●
**Yes**	**22**	●	•	◦	◦			◦	◦	●	●	●	◦		◦	◦	◦	◦	•	◦	•	●	-	◦	◦	•
No	23					○	○		●	○	●	●		○							○		○	-		
No	24					○	○			○	○			○						•	•		●		-	
No	25					●	○			○	○			○							○		●			-

Kolfe Keranyo

	ID	1	2	3	4	5	6	7	8	9	10	11	12	13	14	15	16	17	18	19	20	21	22	23	24	25	26
No. of HIV Services Provided		8	8	15	6	14	14	5	9	5	6	6	7	4	1	9	7	2	4	5	0	10	4	4	10	9	13
Offer Family Planning Services		No	No	**Yes**	**Yes**	**Yes**	**Yes**	No	**Yes**	**Yes**	**Yes**	**Yes**	No	**Yes**	**Yes**	**Yes**	**Yes**	**Yes**	**Yes**	**Yes**	No	**Yes**	**Yes**	**Yes**	No	**Yes**	**Yes**
No	1	-	●	○	○	○	●		○	○	○	○		○	○	○	○	○	○	○		○	○	●		○	●
No	2	●	-	●	●	○	●		○	○	○	○	•	●	○	○	○	○	○	○		○	○	●		○	●
**Yes**	**3**	◦	◦	-	•		•	◦	•				◦		•			●			◦		•	•	◦		●
**Yes**	**4**	◦	◦	•	-	●	●	◦		●	●		◦								◦			•	◦		●
**Yes**	**5**	◦	◦			-		◦				•	◦								◦	•			●		●
**Yes**	**6**	◦	◦	●	•		-	◦					◦								◦			•	◦		●
No	7	●	●	○	○	○	○	-	○	○	●	○		●	○	○	○	○	○	○		○	○	○		○	●
**Yes**	**8**	◦	◦	●				◦	-				◦								◦		●		◦		
**Yes**	**9**	◦	◦		●			◦		-			◦								◦				◦		●
**Yes**	**10**	◦	◦	•	•	●	•	◦		•	-		◦						●		◦	•			●		●
**Yes**	**11**	◦	◦			●		◦				-	◦			●					◦	●			•		●
No	12	•	•	●	○	●	○		●	○	○	○	-	•	○	○	○	○	○	○		○	○	○		○	●
**Yes**	**13**	◦	◦					◦					◦	-							◦				◦		●
**Yes**	**14**	◦	◦	●				◦					◦		-						◦				◦		●
**Yes**	**15**	◦	⊙	•	•	●	●	◦		•		•	◦			-					◦	●			●		●
**Yes**	**16**	◦	◦	●		●	●	◦		•	•	•	◦		•	•	-	•	•		◦	●			●		●
**Yes**	**17**	◦	◦	•				◦					◦					-			◦				◦		●
**Yes**	**18**	◦	◦	•			●	◦			•		◦						-		◦	●			◦		●
**Yes**	**19**	◦	◦					◦			●		◦						●	-	◦				◦		●
No	20			●	○	●	●		○	○	●	○		○	○	○	○	○	●	○	-	○	○	○	●	○	●
**Yes**	**21**	◦	◦			●	•	◦		•			◦						•		•	-			●		●
**Yes**	**22**	◦	◦	●				◦	●				◦		●						◦		-		◦		
**Yes**	**23**	●	●	•				◦			•		●	●					•		◦			-	◦		●
No	24			○	○	●	●		○	○	○	○		○	○	•	○	○	○	○	•	●	○	○	-	○	●
**Yes**	**25**	◦	●	●				●					●								◦		•		◦	-	●
**Yes**	**26**	●	●	●	•	●	●	◦	●				◦	●		•			●		◦			●	◦		-

High	Low	Interactions between HIV and Family Planning Organizations
●	•	Existing interactions between organizations that offer HIV services and/or FP services<br>*High interactions reflect the frrequency of clients referrals from one organization to another at least monthly; low interactions reflect a reported frequency of less than once a month.*

Primary	Secondary	Opportunities for Integration
○	◦	Opportunity for HIV and FP organizations to refer clients to each other<br>*Where "primary" represents an opportunity between an organization that provides HIV services to on that offers FP services*<br>*Where "secondary" represents an opportunity between an organization that provides FP services to on that offers HIV services*
